# On Constipation

**Published:** 1854-11

**Authors:** 


On Constipation. By Professor Trousseau.—Constipation should be defined, a difficulty in the execution of the fœcal matters, independent of any organic lesion. We should not rank among the constipated those in whom defecation is impeded by a tumor or hernia, or invagination, or other mechanical obstacle. Simple constipation is nothing more than absence of alvine evacuations.

Such a condition may depend on many causes. First, there is a sort of phisiological constipation, connected with the constitution of the patient; an individual eats but little, and is endowed with great powers of assimilation. The quantity of excrementitious matters, in such a case, are insufficient to stimulate the in testine. These matters are accordingly retained until they accumulate, and their retention produces uneasiness. There are others, however, who eat largely and assimilate no better than their neighbors, whose intestines are consequently filled with fœcal matters, and still there is no regularity in the dejections. Such individuals suffer from continual malaise; they are in a state of constant discomfort; the want of proper alvine evacuations causes them real suffering.

Defecation, the last stage of digestion, is one of the most complex functions of the economy, and is deranged by a multitude of circumstances. The causes of constipation are numerous, therefore, but the most important are intestine inertia, and the faulty secretion of certain glands. Different secretions predominate in different individuals. One perspires freely, and urinates but little, and secretes but little saliva. The habit of another is the reverse of this; the secerning functions are complementary, as it were, to one another. It is probable that the liver and pancreas also obey this law, and that they are affected by the excessive action of other glands. However hypothetical this proposition may appear, it is as admissible as many others which are received in medicine. It is as rational to suspect the liver and pancreas, when there is a want of lubrification of the bowels, as to suspect the kidneys when there is some derangement of the urine.

Among the causes of constipation, we must examine particularly into the influence of diet, of habit, of senile debility, of diseases foreign to the intestines, and of the abuse of purgatives.

As we have already observed, some individuals eat so little, that the unassimilable matters are insufficient to furnish regular dejections. There are some kinds of food which cause the same result; which are digested with such facility that the fœcal residue amounts to nothing. Rice, for example, disappears almost entirely in digestion, and it is this property which renders it such an appropriate article of nutriment in cases of diarrhœa. Vegetable diet is the least nutricious, and produces the most copious stools. To be convinced of this fact, it is only necessary to compare the dejections of herbiverous and carnivorous animals.

To a certain point, it may be said. that we evacuate the bowels when we wish to do so, only the will must be exercised. If we surmount this inclination to perform this function, it subsides, and may not return for many days. In this way a kind of constipation is produced, which is not the least obstinate or the easiest to cure. It is in this way that so many women, who are prevented from obeying the calls of nature by foolish scruples, and men of letters who are absent-minded or preoccupied, become obstinately constipated.

Purgatives, pills, and powders, irritant enemata and even injections of cold water, and evacuants of all sorts, even when they do not induce a plegmasia of the intestinal canal, deprive it of its normal irritability; it becomes insensible to the simple stimulation which once sufficed to produce the evacuation of the fœcal matters. As tobacco, after provoking an abundant secretion of mucus or saliva by a surexcitation of the glands of the nose and mouth, enfeebles in reality the natural secretion, which diminishes notably when the stimulus is withdrawn, so habitual enemata prevent the intestine from acting by itself, by lowering its excitability. This cause of constipation is very frequent in fashionable women, who early acquire the habit of relieving the bowels by an enema in order to feel unembarrassed during the day. They soon lose the habit of evacuating the bowels naturally, and are forced to have recourse to other remedies of still greater energy.

Child-birth, certain uterine displacements, fissures of the anus, and hemorrhoids, render defecation very painful. Patients in these conditions put off the moment of evacuating the bowels, and induce a secondary constipation, which does not always cease when the cause which produced it is removed.

After several labors, sometimes after one only, the anterior abdominal wall becomes so flaccid and relaxed that it no longer contracts; the intestines are thus deprived of a powerful auxiliary and lose their tonicity.

Age, which impairs all the faculties and all the organic functions, does not spare the intestinal apparatus. Defecation is ill performed in the aged, like digestion and micturition, and retention of the fœces supervenes, without organic disease of the rectum, as retention of urine takes place without organic alteration of the bladder. The intestine undergoes a sort of senile paralysis. It no longer contracts on the excrementitial bolus; it is powerless to expel to expel it; enormous agglomerations of fœces are formed, and dilatations are made above the sphincter ani, which sometimes give rise to grave complications in the operation of lithotomy.

Constipation is a serious disease, and deserves great attention; for even supposing that it is not dangerous in itself, it always complicates the slightest ailment, and frequently lays the foundation of an hypochondria which involves life in numberless disorders.

The treatment should above all be directed to the removal of causes. It must not be thought that constipation is cured when an evacuation is procured; this would be as false as to imagine that a person is cured of wakefulness when he obtains sleep by opiates. We should seek not only to obtain relief for the moment, but regularity in the future. Constipation is not to be cured by Seidlitz powders or other purgatives, which, in the end, only increase it.

In the first place it is necessary to regulate the function; we should do everything to subject it to the empire of the will. We know how great is the power of habit over organic functions. When once accustomed to operate in a certain way, at certain hours, they have a strong tendency to continue to act thus. The habit of going to the privy is like the habit of urinating at certain periods, the habit of shaving, and of washing the face; when once these habits are established, they cannot be dispensed with. A constipated person should visit the water-closet at a convenient hour, he should remain sometime, concentrating all his attention, all his will, all his contractile powers, on the act in question; he should make reiterated and energetic efforts to accomplish it. Whatever the result, he should return at the same hour on the morrow, and if, in the interval, a desire to go to stool is felt, it should be resisted, and the closet should be visited only at the hour chosen.

The diet should be altered. Instead of the abstemious diet often adopted by patients, they should be ordered abundant and nutritious food, of a kind which will at the same time promote digestion and leave a voluminous residue which will force the intestine to disembarrass itself. This disease, we have said, causes numerous disorders. Constipated persons always have oppression of the stomach and head-ache, for which they are ordered a light diet, white meats in small quantity, little wine, etc. This is an error. They should be desired to eat largely of roast meats. They should be compelled to eat, and reminded that, according to the English expression, one leg of mutton follows another.

But sometimes it is necessary to assist the will by an adjuvant, to facilitate an act, the habit of which is lost, as it were. We must beware of purgatives, but yet some medicine is necessary to assist defecation. Belladonna is this remedy *par excellence.* It does not purge; it only facilitates alvine evacuation. To what does it owe this property? It is difficult to say. Perhaps, as opium excites the cutaneous secretion and digitalis that of the urine, so belladonna may influence the liver, pancreas, or other glands opening on the intestinal tract. What is certain, however, is that the fifth of a grain of belladonna almost always procures several alvine evacuations. The dose may be increased to one grain, but as soon as the habit of defecation is restored, the dose of belladonna must be diminished and gradually abandoned.

Suppositories are much used, and commonly succeed well. Country people employ the middle stock of the cabbage; physicians make them of hardened honey or cocoa.

Lastly, if constipation is connected with flaccidity of abdominal walls, we must have recourse to a tight abdominal bandage, which will confine the intestines normally, and enable them to contract. —*Gazette des Hopitaux.*—*Virg. Med. and Surg. Journal.*

